# Perceptions of Interns Toward Integrated Management of Childhood Illness (IMCI) Pre-service Education and Its Impact on Their Clinical Knowledge: A Study at Sultan Qaboos University, Muscat

**DOI:** 10.7759/cureus.69620

**Published:** 2024-09-17

**Authors:** Salama Al Huti, Maisa H Al Kiyumi, Sanjay Jaju, Muna Al Saadoon

**Affiliations:** 1 College of Medicine and Health Sciences, Sultan Qaboos University, Muscat, OMN; 2 Family Medicine and Public Health, Sultan Qaboos University Hospital, University Medical City, Muscat, OMN; 3 Family Medicine and Public Health, College of Medicine and Health Sciences, Sultan Qaboos University, Muscat, OMN; 4 Child Health, College of Medicine and Health Sciences, Sultan Qaboos University, Muscat, OMN

**Keywords:** childhood illness, cross-sectional study, imci, interns, paediatric assessment, sultan qaboos university (squ)

## Abstract

Objectives

The study aims to assess interns' perceptions of Integrated Management of Childhood Illness (IMCI) pre-service education at Sultan Qaboos University (SQU). Specifically, it evaluates how IMCI training during phases 2 and 3 influences interns' clinical practice readiness and knowledge acquisition. The findings will inform evidence-based enhancements to IMCI training programs, ensuring they meet interns' educational needs and optimize clinical skills acquisition.

Methods

This cross-sectional study was conducted at the College of Medicine and Health Sciences (CoMHS) at SQU, Muscat, over a two-month period (September 20, 2023, to November 30, 2023). The questionnaire evaluated interns' sociodemographic factors, perceptions regarding pre-service IMCI training, and IMCI knowledge. IBM SPSS Statistics for Windows, Version 24 (Released 2016; IBM Corp., Armonk, New York) was used to analyze Likert scale responses for frequencies and proportions.

Results

Out of 103 invited interns, 75 participated in the study, resulting in a response rate of 72.8%. Interns who attended three or more IMCI lectures, tutorials, and practical sessions demonstrated a more advanced understanding of IMCI principles compared to those attending fewer sessions. Overall, 63 (84.0%) interns agreed on the effectiveness of IMCI training, 57 (76.0%) interns acknowledged skill enhancement, and 69 (92.0%) interns perceived its practicality for illness assessment. However, only 60.0% (n=45) felt confident in managing sick children. The knowledge assessment revealed varied understanding of IMCI objectives (82.7%, n=62) and components (61.3% (n=46) to 64.0% (n=48)). Clinical case evaluation showed mixed recognition of clinical features and danger signs of childhood illnesses, while awareness of disease preventability through immunization was generally high, except for tuberculosis (74.7%, n=56) and rotavirus (40.0%, n=30).

Conclusion

Interns exhibit positive attitudes towards IMCI principles, demonstrating a strong grasp of related concepts through effective case-based question responses. These results highlight the effectiveness of IMCI training in improving interns' understanding of pediatric healthcare principles, with potential implications for enhancing clinical practice and patient care. Future investigations should explore the impact of IMCI training on interns' clinical practice and patient outcomes.

## Introduction

Children under the age of five are particularly vulnerable to a range of preventable yet potentially fatal diseases, including diarrhea, malaria, and pneumonia, all of which significantly contribute to childhood morbidity and mortality worldwide [1]. Recent data indicate that approximately 2.5 billion cases of diarrhea are reported annually among children under five years of age [2]. Additionally, the World Malaria Report 2020 states that there were 161 million cases of malaria in children under the age of five [1]. Furthermore, an estimated 156 million new cases of pneumonia occur each year in children younger than five years [3].

Regarding childhood-related illnesses in Oman, it is crucial to note the successful eradication of malaria, which was once endemic, over the past several decades [4]. However, despite this achievement, the prevalence of pneumonia and diarrhea among children under five remains a significant concern, with rates of 14 cases per 1000 children and 6 cases per 1000 children, respectively [1]. According to the World Health Organization (WHO), approximately 5.2 million children worldwide died in 2019 before reaching their fifth birthday due to preventable and treatable causes. Notably, while the global under-five mortality rate stands at an estimated 38 deaths per 1000 live births, Oman presents a comparatively lower rate of 10 deaths per 1000 live births, as reported by the United Nations International Children's Emergency Fund (UNICEF) [5].

In this context, implementing the Integrated Management of Childhood Illness (IMCI) strategy emerges as one of the most effective solutions for reducing childhood morbidity and mortality [6]. IMCI, introduced in the mid-1990s by WHO and UNICEF, aims to enhance the overall health of children in developing countries. Its primary objective is to reduce childhood mortality, curtail the incidence of preventable diseases, and foster robust growth in children under five. By addressing the leading causes of childhood morbidity and mortality, this comprehensive program focuses on improving healthcare providers' skills, strengthening healthcare systems, and empowering families and communities to actively engage in their children’s care. Additionally, IMCI promotes the use of clinically validated algorithms to diagnose and treat common childhood illnesses, such as diarrhea, pneumonia, and malaria [6]. Moreover, this strategy is implemented through the establishment of supportive policies and guidelines, the provision of specialized training programs for healthcare professionals, and ensuring the availability of essential medications and supplies [6].

Numerous studies have demonstrated a significant improvement in the global reduction of childhood morbidity and mortality rates due to the implementation of the IMCI strategy. A study conducted in Uganda found that IMCI case management training substantially improved the quality of healthcare provided to children under the age of five [7]. These findings are consistent with similar studies conducted in various countries, including Peru, Brazil, Bangladesh, and Tanzania [7]. In addition, a WHO study revealed that infants who received medical care from IMCI-trained healthcare professionals were more likely to receive an accurate diagnosis. This finding is important as it provides evidence that IMCI-trained healthcare professionals possess greater knowledge and skills for assessing, managing, and treating childhood illnesses [8].

On the other hand, several studies have questioned the overall effectiveness of the IMCI strategy. Amin et al. conducted a comparative study of Pakistani healthcare professionals with and without IMCI training. The study found a disparity in the knowledge and practices of IMCI-trained healthcare professionals regarding the management of childhood illnesses [9]. Similarly, another study conducted to evaluate the knowledge, attitudes, and practices of IMCI-trained physicians revealed that there was no significant difference in their knowledge. These findings can be attributed to several factors, including a lack of interest in implementing the IMCI strategy and the need for additional training workshops [10].

At Sultan Qaboos University (SQU), IMCI was integrated into Phase II lectures in 2007 to familiarize students with its components, childhood disorders, and treatment concepts. This integration continued into Phase III placements at primary healthcare facilities, with IMCI also being included in the summative evaluations of the rotation. Despite these efforts, the literature lacks exploration into the effect of the IMCI strategy on interns, creating a notable gap in knowledge. Therefore, this study aims to assess interns' perceptions of IMCI pre-service education and evaluate the impact of this strategy on their clinical knowledge.

## Materials and methods

This exploratory, cross-sectional, questionnaire-based study aimed to assess interns' perceptions of IMCI pre-service education and its impact on their clinical knowledge. It was conducted at the College of Medicine and Health Sciences (CoMHS) at SQU, Muscat. The study spanned a period of two months during the interns’ internship, starting on September 20, 2023, and ending on November 30, 2023. Inclusion criteria included interns who had graduated from SQU and were currently undergoing their internship at any hospital in Oman. Eligible interns were invited to participate by the co-investigators, who explained the purpose of the study. Written informed consent was obtained, which included information about the study's objectives, justification, and a guarantee of anonymity. Those who agreed to participate were asked to complete the questionnaire.

The self-administered questionnaire consisted of three main sections. The first part included sociodemographic factors such as age, gender, educational level, and the duration of pre-service IMCI training each participant received. The second part assessed interns’ perceptions of pre-service IMCI training and its impact on improving their knowledge, attitudes, and practices (KAP) regarding childhood illnesses, as well as the extent to which it enhanced their skills in managing sick children. This assessment was conducted using an English-language structured questionnaire containing 10 multiple-choice questions (MCQs). The questionnaire was validated by six practicing healthcare experts, and permission to use it was obtained from the corresponding author [11]. The third part was designed specifically to assess the interns' knowledge of IMCI. This section consisted of 10 case-based MCQs covering the main objectives of IMCI, its components, and questions regarding the management of common childhood illnesses such as malaria, pneumonia, and diarrhea, based on their IMCI training. Permission to use this questionnaire was also obtained from the author [12].

The sample size was calculated using the single-proportion absolute-precision finite population correction method. It was assumed that at least 80% of the total eligible participants would have a positive perception of knowledge, attitude, and practice of IMCI pre-service education at SQU. For an absolute precision of 5% and a desired confidence level of 95%, the required sample size for a finite population of 103 interns was calculated to be 73 interns [13].

Statistical analysis

The collected data were analyzed using the IBM SPSS Statistics for Windows, Version 24 (Released 2016; IBM Corp., Armonk, New York). Likert scale responses were presented as frequencies and proportions. The chi-square test of association was employed to assess the significance of differences between specific participant groups. A p-value of <0.05 was considered statistically significant.

## Results

Out of the 103 interns invited to participate in the study, 75 completed the entire questionnaire, resulting in a response rate of 72.8%. Of these respondents, 47 were female (62.7%) and 28 were male (37.3%). The interns' ages ranged from 22 to 27 years, with an average age of 25 years. Regarding the number of IMCI sessions attended, 15 interns (20.0%) attended one lecture/tutorial, 16 (21.3%) attended two lectures/tutorials, 11 (14.7%) attended three or more lectures/tutorials, and 33 (44.0%) attended practical sessions during their junior clerkship.

Upon analysis, the first two groups, consisting of interns who attended one or two lectures/tutorials, were classified as having a basic level of knowledge regarding IMCI. In contrast, the latter two groups, who attended three or more lectures/tutorials or practical sessions, were classified as having an advanced level of knowledge regarding IMCI.

Perception of pre-service IMCI training

In the analysis of question 1, it was found that 63 (84.0%) interns from both the basic and advanced knowledge groups agreed on the effectiveness of IMCI training, while 12 (16.0%) interns from both groups disagreed. For question 2, 57 (76.0%) interns across both groups acknowledged that IMCI training had enhanced their skills, with 38.6% (n=22) from the basic group and 61.4% (n=35) from the advanced group. In contrast, 18 (24.0%) interns disagreed with the statement, comprising 11 (61.1%) from the basic knowledge group and seven (38.9%) from the advanced group.

Regarding question 3, a significant majority, i.e., 69 (92.0%) interns from both knowledge groups, reported that IMCI training offers a practical approach for conducting effective childhood illness assessments, while only six (8.0%) interns disagreed. For question 4, 59 (78.7%) interns from both groups felt that IMCI training improved their clinical knowledge, whereas 16 (21.3%) interns disagreed, with equal representation from each group.

Among the questions surveyed, question 5 had the lowest level of agreement: 45 (60.0%) interns felt confident in managing sick children, with 16 (35.6%) from the basic group and 29 (64.4%) from the advanced group. For question 6, 54 (72.0%) interns agreed that their counseling skills had improved following pre-service IMCI training, while 21 (28.0%) disagreed, with 13 (61.9%) of those from the basic knowledge group. Question 7 showed agreement from the majority-60 interns (80.0%)-indicating that IMCI training helped them conduct holistic assessments of children.

Consistent with the results from question 2, 57 (76.0%) interns in question 8 agreed that pre-service IMCI training was an effective teaching approach, with 63.2% (n=36) from the advanced knowledge group. Conversely, 18 (24.0%) interns disagreed, with 66.7% (n=12) of them from the basic knowledge group. For the final two questions (9 and 10), 55 (73.3%) interns agreed they would apply their IMCI-related skills and knowledge during practice, while 50 (66.7%) agreed they would apply them for parental counseling. Table 1 presents the detailed findings from these two knowledge categories, including results for each of the 10 questions.

**Table 1:** Analysis of Interns’ Responses to IMCI-Related Statements: Group Comparison IMCI: integrated management of childhood illnesses, NS: not significant.

Statements					Response		Knowledge level regarding IMCI training		Total	p-value
							Basic	Advanced		
IMCI training/orientation is an effective teaching method.					Agree		28 (44.4%)	35 (55.6%)	63 (84.0%)	NS 1.000
					Disagree		5 (41.7%)	7 (58.3%)	12 (16.0%)	
Total							33 (100%)	42 (100%)	75 (100%)	
Pre-service IMCI training has enhanced my medical skills.				Agree			22 (38.6%)	35 (61.4%)	57 (76.0%)	NS 0.160
				Disagree			11 (61.1%)	7 (38.9%)	18 (24.0%)	
Total							33 (100%)	42 (100%)	75 (100%)	
Pre-service IMCI training is a practical approach to enhance the evaluation and diagnosis of childhood illnesses.					Agree		29 (42.0%)	40 (58.0%)	69 (92.0%)	NS 0.395
					Disagree		4 (66.7%)	2 (33.3%)	6 (8.0%)	
Total							33 (100%)	42 (100%)	75 (100%)	
Pre-service IMCI training has improved my clinical knowledge.					Agree		25 (42.4%)	34 (57.6%)	59 (78.7%)	NS 0.794
					Disagree		8 (50.0%)	8 (50.0%)	16 (21.3%)	
Total							33 (100%)	42 (100%)	75 (100%)	
Due to pre-service IMCI training, I feel confident in dealing with sick children.						Agree	16 (35.6%)	29 (64.4%)	45 (60.0%)	NS 0.117
						Disagree	17 (56.7%)	13 (43.3%)	30 (40.0%)	
Total							33 (100%)	42 (100%)	75 (100%)	
Pre-service IMCI training has improved my counseling skills.					Agree		20 (37.0%)	34 (63.0%)	54 (72.0%)	NS 0.091
					Disagree		13 (61.9%)	8 (38.1%)	21 (28.0%)	
Total							33 (100%)	42 (100%)	75 (100%)	
Pre-service IMCI training has helped me in conducting a holistic assessment of a child.					Agree		25 (41.7%)	35 (58.3%)	60 (80.0%)	NS 0.601
					Disagree		8 (53.3%)	7 (46.7%)	15 (20.0%)	
Total							33 (100%)	42 (100%)	75 (100%)	
All in all, pre-service IMCI training is an effective teaching approach.	Agree						21 (36.8%)	36 (63.2%)	57 (76.0%)	NS 0.051
	Disagree						12 (66.7%)	6 (33.3%)	18 (24.0%)	
Total							33 (100%)	42 (100%)	75 (100%)	
I apply IMCI-related skills and knowledge during my practice.		Agree					23 (41.8%)	32 (58.2%)	55 (73.3%)	NS 0.713
		Disagree					10 (50.0%)	10 (50.0%)	20 (26.7%)	
Total							33 (100%)	42 (100%)	75 (100%)	
I apply IMCI-related skills for parental counseling.			Agree				20 (40.0%)	30 (60.0%)	50 (66.7%)	NS 0.459
			Disagree				13 (52.0%)	12 (48.0%)	25 (33.3%)	
Total							33 (100%)	42 (100%)	75 (100%)	

Impact of pre-service IMCI training on interns’ clinical knowledge

Interns’ IMCI knowledge was evaluated through 10 case-based MCQs. The first question assessed their understanding of IMCI objectives. It was revealed that 62 (82.7%) interns correctly identified IMCI’s goal of reducing morbidity and mortality among children under five, while 44 (58.7%) were aware of its aim to promote the growth and development of children. For the second question, interns were asked about the components of IMCI, which include improving healthcare workers’ skills, strengthening the health system, and enhancing family and community practices. The results indicated that 46 (61.3%), 48 (64.0%), and 43 (57.3%) of interns were knowledgeable about these components, respectively.

In response to question 3, which focused on the examination of diarrhea and the four essential characteristics to observe, more than two-thirds of interns demonstrated knowledge, with 100% (n=75) identifying the need to check the general condition of the baby. Similarly, a significant majority of interns in question 4 recognized the clinical features of pneumonia, with 94.7% (n=71) correctly identifying an increased respiratory rate as a key indicator.

Regarding question 5, which assessed the essential characteristics for diagnosing anemia, more than half of the interns demonstrated knowledge, with 89.3% (n=67) selecting the examination of the conjunctiva as a diagnostic indicator. Question 6 evaluated interns’ understanding of malnutrition assessment, with all interns opting to assess the degree of malnutrition using the growth chart. However, only 33 (44.0%) selected palm examination as a clinical feature of malnutrition, while the remaining 42 (56.0%) either responded “no” or “I don’t know.”

For the question aimed at assessing interns’ knowledge of diseases preventable by immunization, approximately 90% (n=68) correctly identified diseases such as polio, measles, diphtheria, whooping cough, tetanus, *Haemophilus influenzae* (b), mumps, rubella, pneumococcal infections, and hepatitis B as preventable through immunization. However, recognition of tuberculosis as preventable by immunization was lower, with only 74.7% (n=56) acknowledging it. Additionally, only 40.0% (n=30) of interns were aware that rotavirus infection can be prevented by immunization. A detailed analysis of interns’ responses to IMCI objectives, components, diagnostic assessments of diarrhea, pneumonia, anemia, malnutrition, and vaccine-preventable diseases is further depicted in Table 2.

**Table 2:** Analysis of Interns’ Responses to IMCI Objectives, Components, Diagnostic Assessment of Diarrhea, Pneumonia, Anemia, Malnutrition, and Vaccine-Preventable Diseases IMCI: integrated management of childhood illnesses.

Attributes	Characteristics	Frequency (%)		
		Yes	No	I don’t know
Knowledge regarding IMCI objectives	To reduce morbidity and mortality of under-five children	62 (82.7%)	3 (4.0%)	10 (13.3%)
	To promote growth and development of children through counseling the mothers and caretakers	44 (58.7%)	8 (10.7%)	23 (30.7%)
Knowledge regarding IMCI components	Improving case management skills of health workers	46 (61.3%)	8 (10.7%)	21 (28.0%)
	Improving the health system	48 (64.0%)	5 (6.7%)	22 (29.3%)
	Improving family and community practice	43 (57.3%)	10 (13.3%)	22 (29.3%)
Examination of diarrhea	Level of consciousness	66 (88.0%)	7 (9.3%)	2 (2.7%)
	General condition of the baby	75 (100.0%)	0 (0.0%)	0 (0.0%)
	Skin pinch	65 (86.7%)	9 (12.0%)	1 (1.3%)
	Examination of eyes	54 (72.0%)	17 (22.7%)	4 (5.3%)
Clinical features of pneumonia	Increased respiratory rate	71 (94.7%)	2 (2.7%)	2 (2.7%)
	In-drawing of chest	54 (72.0%)	14 (18.7%)	7 (9.3%)
	Breathlessness	61 (81.3%)	8 (10.7%)	6 (8.0%)
	Cough	58 (77.3%)	9 (12.0%)	8 (10.7%)
Diagnosis of anemia	Examination of conjunctiva	67 (89.3%)	4 (5.3%)	4 (5.3%)
	Examination of nail bed	46 (61.3%)	27 (36.0%)	2 (2.7%)
	Examination of the palm of hand	63 (84.0%)	9 (12.0%)	3 (4.0%)
	Examination of tongue	44 (58.7%)	25 (33.3%)	6 (8.0%)
Examination of malnutrition	Examination of ankle edema	50 (66.7%)	16 (21.3%)	9 (12.0%)
	Assess degree of malnutrition from growth chart	75 (100.0%)	0 (0.0%)	0 (0.0%)
	Examination of the palm of hand	33 (44.0%)	30 (40.0%)	12 (16.0%)
Vaccine-preventable diseases	Tuberculosis	56 (74.7%)	18 (24.0%)	1 (1.3%)
	Polio	74 (98.7%)	0 (0.0%)	1 (1.3%)
	Measles	74 (98.7%)	1 (1.3%)	0 (0.0%)
	Diphtheria	72 (96.0%)	1 (1.3%)	2 (2.7%)
	Whooping cough	73 (97.3%)	1 (1.3%)	1 (1.3%)
	Tetanus	71 (94.7%)	1 (1.3%)	3 (4.0%)
	Hepatitis B	69 (92.0%)	3 (4.0%)	3 (4.0%)
	Mumps	74 (98.7%)	1 (1.3%)	0 (0.0%)
	Rubella	73 (97.3%)	1 (1.3%)	1 (1.3%)
	Pneumococcal (conjugate)	66 (88.0%)	6 (8.0%)	3 (4.0%)
	Hemophilus influenza type (b)	70 (93.3%)	4 (5.3%)	1 (1.3%)
	Rotavirus	30 (40.0%)	29 (38.7%)	16 (21.3%)

**Figure 1:**
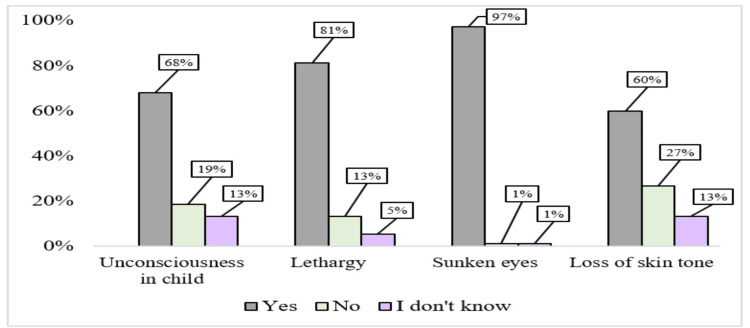
Analysis of Interns’ Responses to Severe Signs of Dehydration

**Figure 2:**
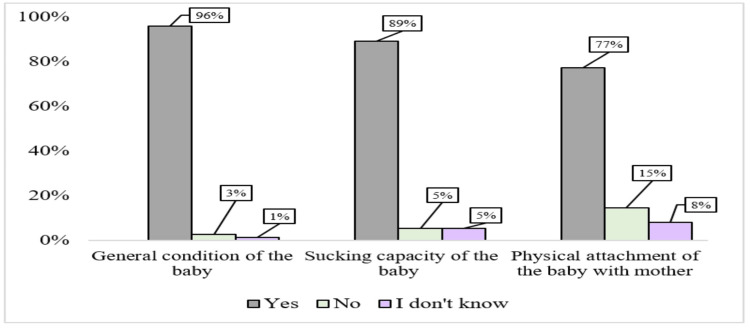
Analysis of Interns’ Responses to Assessment of Breastfeeding

In the analysis of question 7, interns were asked to identify severe signs of dehydration. A significant majority correctly recognized sunken eyes and lethargy as severe indicators, with 97% (n=73) and 81% (n=61) of interns, respectively. However, a lower percentage, 68% (n=51) and 60% (n=45), identified unconsciousness and loss of skin tone as severe signs of dehydration. Regarding question 8, more than two-thirds of interns demonstrated an understanding of important assessment points for breastfeeding, including the general condition of the baby, sucking capacity, and the physical attachment between the baby and the mother. The percentages for these two questions are illustrated in Figures 1, 2.

**Figure 3:**
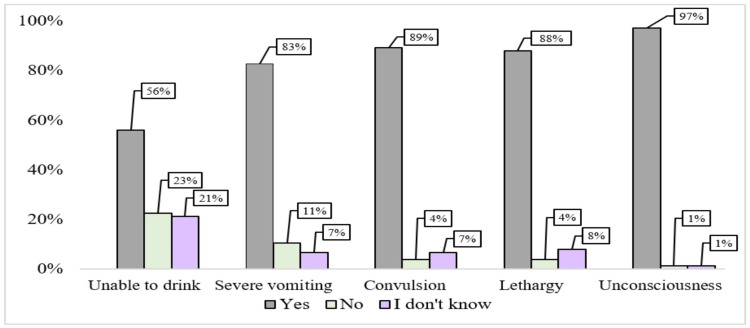
Analysis of Interns’ Recognition of Danger Signs for Childhood Illness

In question 9, interns were asked to identify danger signs for childhood illness. As illustrated in Figure 3, the majority, exceeding 80%, correctly recognized unconsciousness, severe vomiting, convulsions, and lethargy as danger signs. However, there was variation in responses regarding the inability to drink, with 56% (n=42) of interns selecting it as a danger sign, while the remaining 44% (n=33) responded with either "no" or "I don't know."

## Discussion

Highlighting the significance of IMCI pre-service education for interns, the current study indicated a unanimous positive attitude among interns toward IMCI principles and practices. The majority demonstrated a comprehensive understanding of IMCI concepts, as evidenced by their performance on IMCI case-based questions.

Delving into more specific findings regarding interns’ perceptions, despite variations in the number of IMCI lectures/tutorials attended, most interns emphasized the importance of IMCI training in enhancing their clinical skills and medical knowledge. Many interns expressed a willingness to apply IMCI skills and knowledge in medical practice and the assessment of children’s illnesses. However, two questions elicited less agreement, particularly those assessing interns’ confidence in managing sick children and their proficiency in parental counseling skills.

A recent study conducted on medical students at SQU, who later became interns and received the same IMCI pre-service education, sheds light on potential factors contributing to interns’ diminished agreement regarding the impact of IMCI training on their parental counseling and managing sick children. These factors include irregular attendance in IMCI lectures, inadequate training, and time constraints [14].

The relationship between interns’ attendance at IMCI lectures and their perceptions was also investigated. Interns who attended three or more IMCI lectures/tutorials or practical sessions during junior clerkship tended to demonstrate higher rates of agreement with IMCI-related statements compared to those who attended only one or two lectures/tutorials. Although not statistically significant, this suggests a potential association between attendance frequency and interns’ perceptions of IMCI.

Moving on to the second objective of this study, the assessment of interns’ clinical knowledge regarding IMCI revealed their proficiency across various domains. The majority demonstrated a solid understanding of IMCI objectives, components, and case-based scenarios, including conditions such as diarrhea, pneumonia, anemia, dehydration, malnutrition, childhood danger signs, breastfeeding, and vaccine-preventable diseases. However, a notable gap was observed in their recognition of palm examination as a sign of malnutrition and the preventability of rotavirus through immunization. These findings may be attributed to differences in interns’ exposure to certain IMCI concepts. Further investigation is needed to understand the exact reasons behind these gaps and to improve IMCI training accordingly.

The overall findings of this study align with similar studies in the literature. A recent study conducted by Al-Yahyahi, which focused on medical students now serving as interns in our study, showcased positive perceptions among SQU medical students regarding IMCI pre-service education and their readiness to apply the acquired clinical knowledge and skills in future practice [14]. Another study by Al-Abri, involving the same group of medical students, demonstrated a strong understanding of the clinical practice implications of IMCI pre-service training [15]. Our findings emphasize that SQU graduates retain their knowledge and positive perceptions of IMCI training.

Research suggests that children treated by IMCI-trained providers are more likely to receive appropriate care, with these providers also demonstrating enhanced communication skills with caregivers [16]. These results align with a study by Al-Araimi, which indicated that IMCI-trained healthcare workers provided holistic care to sick children [8]. Nevertheless, it is important to consider the potential variations in healthcare settings that could influence the effectiveness of IMCI implementation [17].

Few studies have assessed the impact of IMCI training on healthcare workers' proficiency in identifying and managing cases. A study in India reported better performance by IMCI-trained medical students in detecting and managing major childhood illnesses compared with an untrained group [18]. Similarly, another study revealed that IMCI training for medical students significantly improved their abilities in case identification, accurate clinical diagnosis, and decision-making regarding referrals [19].

In contrast, a study conducted in Egypt involving 157 physicians found that the IMCI strategy encountered limited acceptance among doctors, primarily due to inadequate training, insufficient supervision, and high patient-to-physician ratios [20]. Additionally, a study in Pakistan, which included 57 physicians, identified gaps in both knowledge and practices among IMCI-trained doctors [9]. Correspondingly, a study conducted on 237 healthcare workers in Bangladesh reported that nearly half of the respondents had poor knowledge regarding IMCI [12].

The findings of the current study are significant as they underscore the importance of IMCI pre-service education for interns, illustrating its direct impact on their clinical skills and knowledge. To the best of the authors' knowledge, previous studies have not explored interns’ perceptions of IMCI training and its impact on their clinical knowledge at both regional and global levels. Therefore, this study is important for promoting the implementation of IMCI training in Oman and contributes to filling this gap in the literature.

Limitations

This study is subject to certain limitations. First, due to the limited number of participants, the results cannot be generalized. Second, the sample was drawn from a single educational center, which may introduce selection bias. Third, while the statistical results suggest that IMCI-trained interns express intentions to apply their acquired skills in clinical practice, further investigations are needed to validate these intentions. Lastly, interns received their IMCI training during phases 2 and 3, which may lead to recall bias, as participants might have difficulty remembering details of their IMCI training.

Recommendations 

In future research, it is recommended that more studies should be done assessing different educational centers with a larger sample size for comparative results. Additionally, it is important to examine various methods of teaching IMCI and their impact on interns’ patient care. Moreover, keeping interns updated on IMCI principles and practices will help improve their clinical skills.

## Conclusions

This study demonstrated that interns generally have a positive attitude toward IMCI principles and practices. Most interns showed a solid understanding of IMCI concepts, as reflected in their ability to effectively answer IMCI case-based questions. These findings highlight the effectiveness of IMCI training in enhancing interns' understanding of pediatric healthcare principles and suggest its potential to improve clinical practice and patient care. Further research is needed to explore the long-term impact of IMCI training on interns’ clinical practice and patient outcomes.
